# Supplementary material for: A comparison of penalised regression methods for informing the selection of predictive markers
Source: PLoS One. 2020 Nov 20;15(11):e0242730. doi: 10.1371/journal.pone.0242730 (PMC7678959; doi:10.1371/journal.pone.0242730)
Supplement: S1 Table — Note: a = approach to combine repeated measures data; SR = Self report, PR = Parent report; SMFQ = Short Mood and Feelings Questionnaire [39], RBPC = Revised Behaviour Problem Checklist [40], RCMAS = Revised Children’s Manifest Anxiety Scale [41], SRED = Self-Report Early Delinquency Instrument [42], SSRS = Social Skills Rating System [43], CSEI = Coopersmith Self-Esteem Inventory [44], PIES = Psychosocial Inventory of Ego Strengths [45], ACER SLQ = ACER School Life Questionnaire [46], OSBS = O’Donnell School Bonding Scale [47], IPPA = Inventory of Parent and Peer Attachment [48], CBQ = Conflict Behaviour Questionnaire [49], FACES II = Family Adaptability and Cohesion Evaluation Scale [50], RAS = Relationship Assessment Scale [51], OHS = Overt Hostility Scale [52], ZTAS = Zuckerman’s Thrill and Adventure Seeking Scale [53], FFPQ = Five Factor Personality Questionnaire [54], GBFM = Goldberg’s Big Five Markers [55], SATI = School Age Temperament Inventory [56], more information on ATP derived scales can be found in Vassallo and Sanson [25]. (DOCX) [file pone.0242730.s001.docx]

S1 Table. Description of adolescent indicators

| Construct | Scale/Scoring | Respondent | 13-14 | 15-16 | 17-18 | Cronbach's α | Multi-wave approach ^a^ |
| --- | --- | --- | --- | --- | --- | --- | --- |
| **Individual Factors** |  |  |  |  |  |  |  |
| Biological |  |  |  |  |  |  |  |
| Sex | 1=Female | PR |  |  |  | - |  |
| Ethnicity | 1=Caucasian | PR |  |  |  | - |  |
| Height | cms | SR |  | x |  | - |  |
| Weight | kgs | SR |  | x |  | - |  |
| Birth Order | Order of birth | PR |  |  | x | - |  |
| Puberty | 1=Menstruation/voice breaking | SR | x |  |  | - |  |
| Internalising/Externalising |  |  |  |  |  |  |  |
| Depression | SMFQ | SR | x | x | x | 0.82, 0.86, 0.87 | Mean |
| Eating Disorder | EDI | SR |  | x |  | 0.90 |  |
| Hyperactivity | RBPC | SR | x | x | x | 0.76, 0.75, 0.76 | Mean |
| Oppositional | RBPC | SR | x | x | x | 0.66, 0.70, 0.65 | Mean |
| Anxious-Fearful | RBPC, RCMAS | SR | x | x | x | 0.71, 0.84, 0.83 | Mean |
| Conduct Disorder | RBPC | PR | x | x | x | 0.91, 0.91, 0.91 | Mean |
| Socialised Aggression | RBPC | PR | x | x | x | 0.84, 0.88 0.86 | Mean |
| Attention Problems | RBPC | PR | x | x | x | 0.89, 0.90, 0.90 | Mean |
| Anxiety/Withdrawal | RBPC | PR | x | x | x | 0.85, 0.87, 0.88 | Mean |
| Psychotic Behaviour | RBPC | PR | x | x | x | 0.59, 0.63, 0.66 | Mean |
| Motor Tension/Excess | RBPC | PR | x | x | x | 0.72, 0.71, 0.75 | Mean |
| Delinquency | SRED | SR | x | x | x | 0.83, 0.82, 0.84 | Mean |
| Personality and Temperament |  |  |  |  |  |  |  |
| Overall temperament | ATP derived | PR | x | x | x | - | Mean |
| Negative Reactivity | SATI | PR | x | x | x | 0.92, 0.92, 0.84 | Mean |
| Persistence | SATI | PR | x | x | x | 0.92, 0.92, 0.61 | Mean |
| Approach | SATI | PR | x | x | x | 0.89, 0.88, 0.85 | Mean |
| Activity | SATI | PR | x | x |  | 0.78, 0.78 | Mean |
| Sensation Seeking | ZTAS | SR |  | x |  | 0.72 |  |
| Openness | FFPQ, GBFM | SR |  | x | x | 0.72, 0.65 | Mean |
| Conscientiousness | FFPQ, GBFM | SR |  | x | x | 0.72, 0.76 | Mean |
| Extraversion | FFPQ, GBFM | SR |  | x | x | 0.69, 0.74 | Mean |
| Agreeableness | FFPQ, GBFM | SR |  | x | x | 0.66, 0.70 | Mean |
| Neuroticism | FFPQ, GBFM | SR |  | x | x | 0.75, 0.70 | Mean |
| Social Competence |  |  |  |  |  |  |  |
| Assertiveness | SSRS | SR | x | x |  | 0.65, 0.78 | Mean |
| Cooperation | SSRS | SR | x |  |  | 0.75 |  |
| Empathy | SSRS | SR | x |  |  | 0.81 |  |
| Self-Control | SSRS | SR | x |  |  | 0.68 |  |
| Responsibility | SSRS | PR | x | x |  | 0.72, 0.48 | Mean |
| Emotional Control | ATP derived | SR |  | x | x | 0.68, 0.56 | Mean |
| Self Esteem | CSEI | SR |  |  | x | 0.76 |  |
| The Future: Optimism | Adapted PIES | SR |  |  | x | 0.79 |  |
| The Future: Identity Clarity | Adapted PIES | SR |  |  | x | 0.84 |  |
| The Future: Desire For Privacy | Adapted PIES | SR |  |  | x | 0.67 |  |
| The Future: Readiness For Intimacy | Adapted PIES | SR |  |  | x | 0.63 |  |
| Positive Development |  |  |  |  |  |  |  |
| Civic engagement | ATP derived | SR |  | x |  | 0.66 |  |
| Political engagement | ATP derived | SR |  | x |  | 0.78 |  |
| Volunteering | 1=volunteer | SR |  |  | x | - |  |
| Community Bonding | ATP derived | SR |  |  | x | 0.79 |  |
| Political Awareness | ATP derived | SR |  |  | x | 0.77 |  |
| Environmental Awareness | ATP derived | SR |  |  | x | 0.73 |  |
| Community/Family Orientation | ATP derived | SR |  |  | x | 0.63 |  |
| Personal Success Orientation | ATP derived | SR |  |  | x | 0.47 |  |
|  |  |  |  |  |  |  |  |
| **Relational Factors** |  |  |  |  |  |  |  |
| Peer and Family Relationships |  |  |  |  |  |  |  |
| Peer Attachment | IPPA | SR | x |  | x | 0.77, 0.86 | Mean |
| Supportive Friendships | ATP derived | SR | x | x |  | 0.85, 0.90 | Mean |
| Deviant Peers | ATP derived | SR | x | x |  | 0.87, 0.87 | Mean |
| Peer Involvement | ATP derived | PR | x |  |  | 0.83 |  |
| Group Participation | ATP derived | PR | x |  |  | 0.66 |  |
| Parent Relationship | IPPA | SR | x |  | x | 0.86, 0.89 | Mean |
| Parent teen conflict | CBQ | PR |  |  | x | 0.88 |  |
| Parent teen positive relationship | CBQ | PR |  |  | x | 0.81 |  |
| Family cohesion | FACES II | PR |  |  | x | 0.88 |  |
| Parent Marital Status | 1=experienced separation | PR | x | x | x | - | Max |
| Parent Marital Conflict | Adapted RAS & OHS | PR |  |  | x | 0.83, 0.82 |  |
| Parent Marital Closeness | Adapted RAS & OHS | PR |  |  | x | 0.95, 0.96 |  |
| Parenting Practices |  |  |  |  |  |  |  |
| Warmth | ATP derived | PR | x | x | x | 0.78, 0.81, 0.91 | Mean |
| Inductive Reasoning | ATP derived | PR | x | x |  | 0.74, 0.78 | Mean |
| Monitoring | ATP derived | PR | x | x |  | 0.49, 0.63 | Mean |
| Punishment | ATP derived | PR | x | x |  | 0.72, 0.70 | Mean |
|  |  |  |  |  |  |  |  |
| **Contextual factors** |  |  |  |  |  |  |  |
| Mother education Level | 1=post high-school | PR | x | x | x | - | Max |
| Father education Level | 1=post high-school | PR | x | x | x | - | Max |
| Unemployment | 1=unemployed | PR | x | x | x | - | Max |
| Stressful Life Events | Number of negative events | PR | x | x | x | - | Mean |
| Child Living Status | 1=not living with bio parents | PR | x | x | x | - | Max |
| House Moves | Number of house moves | PR | x | x | x | - | Max |
| Family Size | Number of children | PR | x |  | x | - | Max |
| Death of Parent | 1=death | SR/PR |  |  | x | - |  |
| Financial Stress | No, a little, a lot | PR |  |  | x | - |  |
| School and Work |  |  |  |  |  |  |  |
| School Problems | ATP derived | SR | x |  |  | 0.70 |  |
| School Changes | Number of school changes | PR | x | x |  | - | Max |
| Positive Effect Towards School | ACER SLQ | SR |  | x |  | 0.86 |  |
| Relationship With Teachers | ACER SLQ | SR |  | x |  | 0.83 |  |
| Provides Prestige | ACER SLQ | SR |  | x |  | 0.82 |  |
| Long Term Relevance | ACER SLQ | SR |  | x |  | 0.82 |  |
| Confidence In Ability/Success | ACER SLQ | SR |  | x |  | 0.83 |  |
| School Bonding | OSBS | SR |  |  | x | 0.88 |  |
| Disciplinary Trouble | 1=disciplined/suspended/expelled | PR |  | x |  | - |  |
| Currently Working | 1=working | SR/PR |  | x | x | - | Max |
| Currently Studying | 1=studying | SR |  |  | x | - |  |
|  |  |  |  |  |  |  |  |
| **Substance Use Factors** |  |  |  |  |  |  |  |
| Alcohol Frequency | Days per month | SR | x | x | x | - | Mean |
| Binge Drinking Frequency | Days per month | SR |  | x | x | - | Mean |
| Tobacco Frequency | Days per month | SR | x | x | x | - | Mean |
| Cannabis Frequency | Days per month | SR |  | x | x | - | Mean |
| Illicit Use | Monthly use | SR | x | x | x | - | Max |
| Alcohol Problems | 1=alcohol problems | SR |  | x | x | - | Max |
| Cannabis Problems | 1=cannabis problems | SR |  |  | x | - |  |
| Illicit Problems | 1=illicit problems | SR |  |  | x | - |  |
| Substance Use Environment |  |  |  |  |  |  |  |
| Parent alcohol Use | 1=alcohol user | PR | x |  | x | - | Max |
| Parent tobacco Use | 1=tobacco user | PR | x |  | x | - | Max |
| Adolescent drinks alcohol at home | 1=drinks at home | PR |  |  | x | - |  |
| Adolescent allowed alcohol at parties | 1-takes alcohol to parties | PR |  |  | x | - |  |
| Worry about child’s drinking | No, a little, a lot | PR |  |  | x | - |  |
| Talk with teen about drinking | No, a little, a lot | PR |  |  | x | - |  |
| Adolescent smokes at home | 1=smokes at home | PR |  |  | x | - |  |
| Adolescent smokes with parent | 1=smokes with parent | PR |  |  | x | - |  |
| Worry about child’s smoking | No, a little, a lot | PR |  |  | x | - |  |
| Talk with teen about smoking | No, a little, a lot | PR |  |  | x | - |  |
| Note: a = approach to combine data across multiple waves, SR=Self report, PR=Parent report;  SMFQ=Short Mood and Feelings Questionnaire (Angold et al., 1995), RBPC=Revised Behaviour Problem Checklist (Quay & Peterson, 1993), RCMAS=Revised Children’s Manifest Anxiety Scale (Reynolds & Richmond, 1997), SRED=Self-Report Early Delinquency Instrument (Moffitt & Silva, 1988), SSRS=Social Skills Rating System (Gresham & Elliott, 1990), CSEI=Coopersmith Self-Esteem Inventory (Coopersmith, 1982), PIES=Psychosocial Inventory of Ego Strengths (Markstrom, Sabino, Turner, & Berman, 1997), ACER SLQ=ACER School Life Questionnaire (Ainley, Reed, & Miller, 1986), OSBS=O’Donnell School Bonding Scale (O’Donnell, Hawkins, & Abbott, 1995), IPPA=Inventory of Parent and Peer Attachment (Armsden & Greenberg, 1987), CBQ=Conflict Behaviour Questionnaire (Prinz, Foster, Kent, & O’Leary, 1979), FACES II=Family Adaptability and Cohesion Evaluation Scale (Olson, Portner, & Bell, 1982), RAS=Relationship Assessment Scale (Hendrick, 1988), OHS=Overt Hostility Scale (Porter & Leary, 1980), ZTAS=Zuckerman’s Thrill and Adventure Seeking Scale (Zuckerman, Kolin, Price, & Zoob, 1964), FFPQ= Five Factor Personality Questionnaire (Lanthier & Bates, 1995), GBFM=Goldberg’s Big Five Markers (Goldberg, 1992), SATI=School Age Temperament Inventory (McClowry, 2016), more information on ATP derived scales can be found in Vassallo & Sanson (2013). | | | | | | | |
